# Regulated expression of a transgene introduced on an oriP/EBNA-1 PAC shuttle vector into human cells

**DOI:** 10.1186/1472-6750-9-88

**Published:** 2009-10-16

**Authors:** Hanne A Askautrud, Elisabet Gjernes, Gro L Størvold, Mona M Lindeberg, Jim Thorsen, Hans Prydz, Eirik Frengen

**Affiliations:** 1 Department of Medical Genetics, Ullevål University Hospital and Faculty of Medicine, University of Oslo, Oslo, Norway; 2 Biotechnology Centre of Oslo, University of Oslo, Oslo, Norway; 3 Aquaculture Protein Center, Department of Basic Sciences and Aquatic Medicine, Norwegian School of Veterinary Science, Oslo, Norway

## Abstract

**Background:**

Sequencing of the human genome has led to most genes being available in BAC or PAC vectors. However, limited functional information has been assigned to most of these genes. Techniques for the manipulation and transfer of complete functional units on large DNA fragments into human cells are crucial for the analysis of complete genes in their natural genomic context. One limitation of the functional studies using these vectors is the low transfection frequency.

**Results:**

We have constructed a shuttle vector, pPAC7, which contains both the *EBNA-1 *gene and *ori*P from the Epstein-Barr virus allowing stable maintenance of PAC clones in the nucleus of human cells. The pPAC7 vector also contains the *EGFP *reporter gene, which allows direct monitoring of the presence of PAC constructs in transfected cells, and the *Bsr*-cassette that allows highly efficient and rapid selection in mammalian cells by use of blasticidin. Positive selection for recombinant PAC clones is obtained in pPAC7 because the cloning sites are located within the SacBII gene. We show regulated expression of the *CDH3 *gene carried as a 132 kb genomic insert cloned into pPAC7, demonstrating that the pPAC7 vector can be used for functional studies of genes in their natural genomic context. Furthermore, the results from the transfection of a range of pPAC7 based constructs into two human cell lines suggest that the transfection efficiencies are not only dependent on construct size.

**Conclusion:**

The shuttle vector pPAC7 can be used to transfer large genomic constructs into human cells. The genes transferred could potentially contain all long-range regulatory elements, including their endogenous regulatory promoters. Introduction of complete genes in PACs into human cells would potentially allow complementation assays to identify or verify the function of genes affecting cellular phenotypes.

## Background

Sequencing of the human genome has led to most genes being available in F-plasmid based and P1-derived bacterial artificial chromosomes (BACs/PACs) [1-5]. Even though the genome projects have resulted in the discovery of a large number of genes, limited functional information has been assigned to most of these genes. One approach to analyze the biological functions of genes is direct introduction of complete genes into appropriate cells to monitor the phenotypic effects of the gene or its mutated versions. The average mammalian gene has been estimated to span 50 kb, and sequences distant from the gene itself are often important for proper developmental and tissue-specific expression [6]. Furthermore, constructs containing complete genes including introns have shown stable expression and facilitate physiological expression levels and tissue-specific expression patterns [7,8]. Therefore, techniques for the manipulation and transfer of complete functional units on large DNA fragments into human cells are crucial for the analysis of complete genes in their natural genomic context.

Plasmid vectors based on EBV are stably maintained extrachromosomally over long periods of time, although segregation of the vectors to daughter cells upon cell division is random rather than equal [9,10]. The functional components of such vectors are the EBV latent origin of replication (*ori*P) [11], which interacts with the viral transactivator protein EBNA-1 [12] to promote episomal maintenance. Functional studies using BACs have been performed in hamster cells [13], transgenic mice [14,15], and in human cells [16]. BAC vectors with *ori*P/EBNA-1 have successfully been used for introduction of large transgenes into mammalian cells and several studies have demonstrated the general utility of the *ori*P/EBNA-1 system in mammalian cells [9,16-27].

We have constructed the pPAC7 shuttle vector to facilitate the use of large-insert bacterial clones (BACs/PACs) as *ori*P/EBNA-1 episomes for functional analysis of complete genes in human cell lines. Our vector carries the Enhanced Green Fluorescent Protein (*EGFP*) reporter gene, which allows direct monitoring of transfected cells for the presence of PACs. We document regulated expression of the *CDH3 *gene carried as a 132 kb genomic insert cloned into pPAC7, demonstrating that the pPAC7 vector can be used for functional studies of genes in their natural genomic context. Even though the BAC/PAC vectors can be used to introduce large genomic fragments into human cells, the large size of these DNA constructs has provided challenges to their use as experimental or therapeutic materials. One of these challenges is the inefficient transfection of large DNA constructs. When introducing constructs ranging in size from 23,4 kb to 155 kb into human cell lines a direct relationship between construct size and transfection efficiency was not detected.

## Results & Discussion

### The functionality of the shuttle vector pPAC7

**Figure 1:**
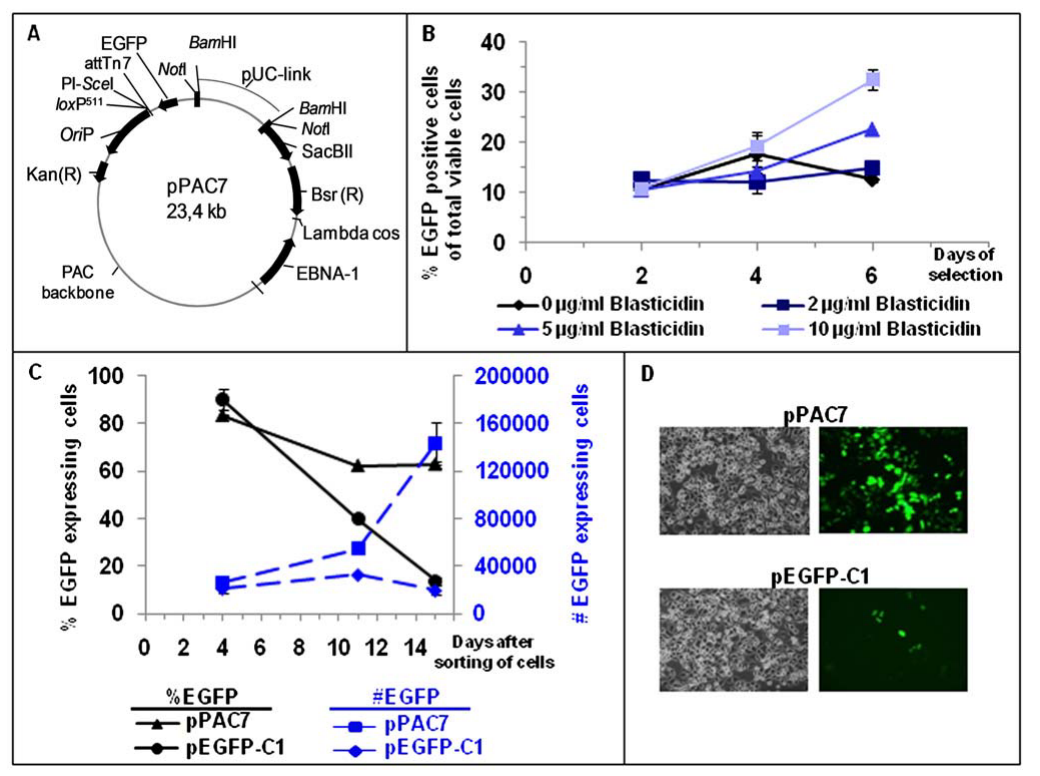
**The pPAC7 vector**. A) Schematic map of the pPAC7 vector which contains the *EGFP *gene cassette (EGFP), the blasticidin deaminase gene cassette (Bsr), the *EBNA-1 *gene cassette (EBNA-1), and the Epstein Barr virus origin of replication (*ori*P). The pUC-link fragment is removed by *BamH*I or *Not*I digestion during the cloning process and replaced by large genomic DNA fragments allowing positive selection for recombinant clones on sucrose containing media due to the *Sac*BII gene present on the vector. The kanamycin resistance gene (Kan(R)) is a bacterial selection marker. The *Not*I restriction sites are used when verifying the different constructs. B) SK-BR-3 cells were transfected with pPAC7 and grown in the presence of blasticidin using the concentrations indicated. The percent of EGFP positive cells, from all viable cells, were measured by flow cytometry after 2, 4 and 6 days. The experiments were performed in triplicates with standard deviations shown. C) SK-BR-3 cells transfected with pPAC7 or pEGFP-C1 were sorted by flow cytometry and grown in non-selective medium. The percentage and total number of EGFP expressing cells was measured by flow cytometry 4, 11 and 15 days after cell sorting. The experiments were performed in triplicates with standard deviations indicated. D) The fluorescent microscopy pictures show the SK-BR-3 cells transfected with pEGFP-C1 and pPAC7 15 days after cell sorting. The shutter time is 1306 ms for pEGFP-C1 transfected and 390 ms for pPAC7 transfected cells.

A detailed description of the vector pPAC7 [GenBank: FJ710109] is given in the Methods section, and a map of the vector is shown in Figure 1A. The pUC-link fragment in pPAC7, which separates the *Sac*BII gene from its promoter is removed in the PAC construction process. The *Sac*BII gene product converts sucrose to levan, which is toxic to the host cells. During PAC construction, large DNA fragments replace the stuffer fragment maintaining the separation of the promoter and the coding region of the *Sac*BII gene, thus resulting in positive selection for recombinant PAC clones [28]. pPAC7 also contains the *att*Tn7 sequence, permitting highly specific Tn7 mediated insertion of sequence elements into the vector portion of the pPAC7 vector for tailor-made functional analysis of genes carried on the cloned inserts [28]. Specific linearization of the PACs can be achieved by using the lambda terminase or the PI-SceI enzyme cleaving in the vector cos site or PI-SceI respectively [28].

In order to verify the functionality of the elements included in pPAC7, the vector was transfected into the cell line SK-BR-3. The increasing fraction of viable EGFP expressing cells, that was obtained when grown in media containing increasing concentrations of blasticidin, demonstrated the presence of functional *Bsr *and *EGFP *gene cassettes in pPAC7 (Figure 1B). SK-BR-3 cells transfected with either pEGFP-C1 or pPAC7 were sorted by flow cytometry for EGFP, resulting in a cell population containing 95% EGFP expressing cells. The percentage of EGFP expressing cells remained high and the number of EGFP positive cells increased when SK-BR-3 cells transfected with pPAC7 were grown in non-selective medium (Figure 1C). Control cells transfected with a non-replicating vector, pEGFP-C1, showed no increase in the number of EGFP positive cells (Figure 1C). The fact that the pPAC7 vector is being replicated and distributed to daughter cells demonstrates the funtionality of the *Ori*P/EBNA-1 system. The high number of EGFP positive cells after transfection with pPAC7 was also visualized by microscopy 15 days after sorting and growth in non-selective medium (Figure 1D). Note that not only are more cells positive for EGFP after transfection with pPAC7, but the fluorescence intensity is three fold lower in pEGFP-C1 transfected cells (camera shutter time: 1306 ms) compared to pPAC7 transfected cells (camera shutter time: 390 ms). The difference in intensities may indicate the presence of variable copy numbers of pPAC7, which is expected for *ori*P/EBNA-1 episomes [9,17]. Also *Ori*P is known to act as an transcriptional enhancer in the presence of the EBNA-1 protein, which also can account for the increased fluorescence in the pPAC7 transfected cells [29]. In summary, these results verify that the *Bsr*, *EGFP*, *ori*P and *EBNA-1 *elements are functional in the pPAC7 vector.

### Regulated expression of the *CDH3 *gene from an insert in pPAC7

**Figure 2:**
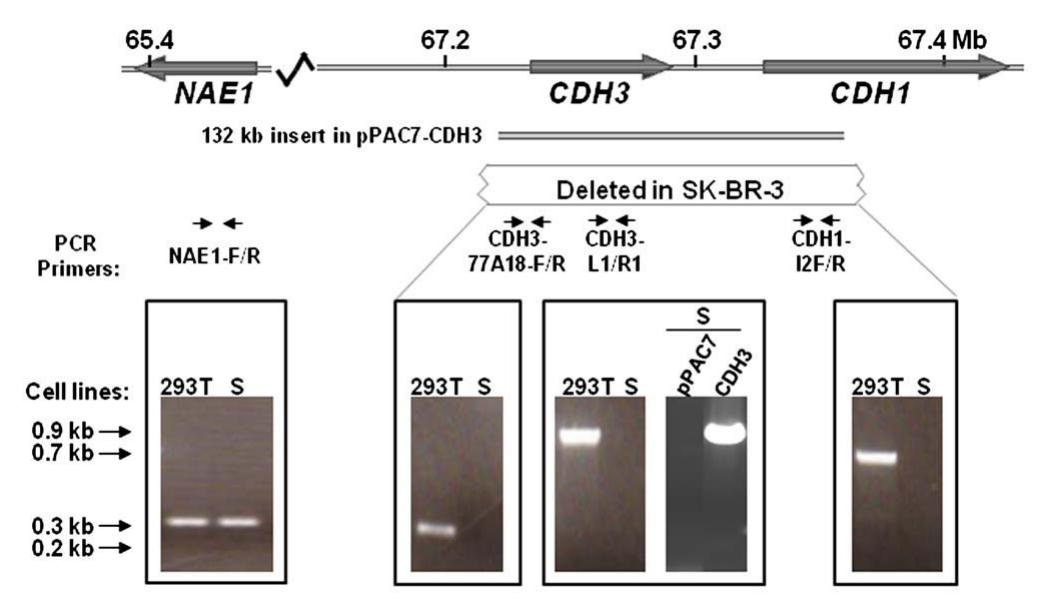
**Stable maintenance of pPAC7 in SK-BR-3 cells**. Schematic presentation of the *CDH3 *and *CDH1 *genes with the positions on human chromosome 16q22.1 shown in Mb. The 132 kb insert cloned in pPAC7-CDH3, and the positions of the PCR primers used are indicated. DNA isolated from the 293T, SK-BR-3 (S), SK-BR-3/pPAC7 (S-pPAC7) and SK-BR-3/pPAC7-CDH3 (S-CDH3) cell lines was amplified by PCR using the primers indicated [see Additional file 2]. In these experiments the control primers in the *NAE1 *gene gave a PCR product both from SK-BR-3 and 293T, while no products were detected from SK-BR-3 using the 3 primer pairs from the *CDH1*-*CDH3*-region. Selected fragments in the GeneRuler™ 100 bp DNA Ladder (Fermentas, MD, USA) are indicated.

It has previously been shown that both alleles of the *CDH1 *gene is deleted in the SK-BR-3 cell line [30]. We demonstrate that the deletion extends more than 100 kb upstream of *CDH1*, covering all the exons of *CDH3 *and at least 10 kb upstream of this gene (Figure 2). Since the SK-BR-3 cell line is completely lacking the *CDH3 *gene, and CDH3 has been shown to be regulated by steroids [31], we chose to use this gene to demonstrate regulated gene expression from the pPAC7 vector. In order to express the *CDH3 *gene within its native genomic context, the complete gene, including all exons, introns and 32 kb upstream sequence was transferred as a 132 kb fragment into pPAC7 (see Methods). This construct, pPAC7-CDH3, was transfected into SK-BR-3 and the cells grown in selective medium for 6 weeks. The resulting cell population, SK-BR-3/pPAC7-CDH3, carried the *CDH3 *fragment (Figure 2, see additional file 1 for list of primers). Because pPAC7 replicates (Figure 1), we assume that the construct pPAC7-CDH3 is maintained as an episome in this cell population.

**Figure 3:**
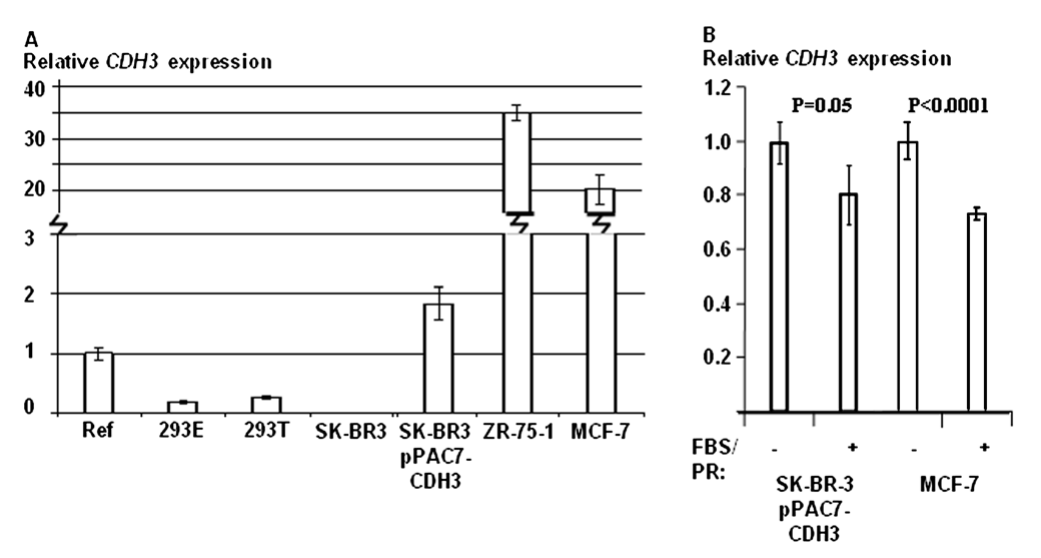
**Regulated *CDH3 *expression from pPAC7-CDH3 in SK-BR-3**. A) Real Time PCR experiments showing relative endogenous *CDH3 *expression in selected cell lines. B) The *CDH3 *expression levels in the cell population SK-BR-3/pPAC7-CDH3 (*CDH3 *transgene) and in MCF-7 (endogenous) are decreased when grown in regular media (FBS/PR) (+) compared to the cells grown media without phenol red containing charcoal stripped FBS (-).

To verify transcription of CDH3 in the SK-BR-3/pPAC7-CDH3 cell population we performed Real Time PCR (Figure 3A). As expected, the parent SK-BR-3 cell line showed no endogenous *CDH3 *expression. Since the CDH3 transcript was detected in the SK-BR-3/pPAC7-CDH3 cells using an assay which spans sequences in exon 12, 13 and 14, this strongly suggests that correct transcription and splicing of *CDH3 *was obtained in these cells.

When the breast cancer cell line MCF-7 is grown in media with charcoal stripped FBS and without phenol red, a clear increase in *CDH3 *expression is observed compared to cells grown in regular medium (Figure 3B). This difference in expression can most probably be explained by the presence of steroids in the latter medium, which are known to suppress *CDH3 *expression [31]. Decreased *CDH3 *expression was also detected when SK-BR-3/pPAC7-CDH3 cells were grown in media containing phenol red and regular FBS (Figure 3B). In total, these results demonstrate regulated *CDH3 *expression when the pPAC7-CDH3 construct carrying the complete gene is introduced into the human cell line SK-BR-3. Thus, constructs in pPAC7 provide important tools in functional experiments, including the identification and validation of candidate disease genes.

### Transfection efficiency and construct size

**Figure 4:**
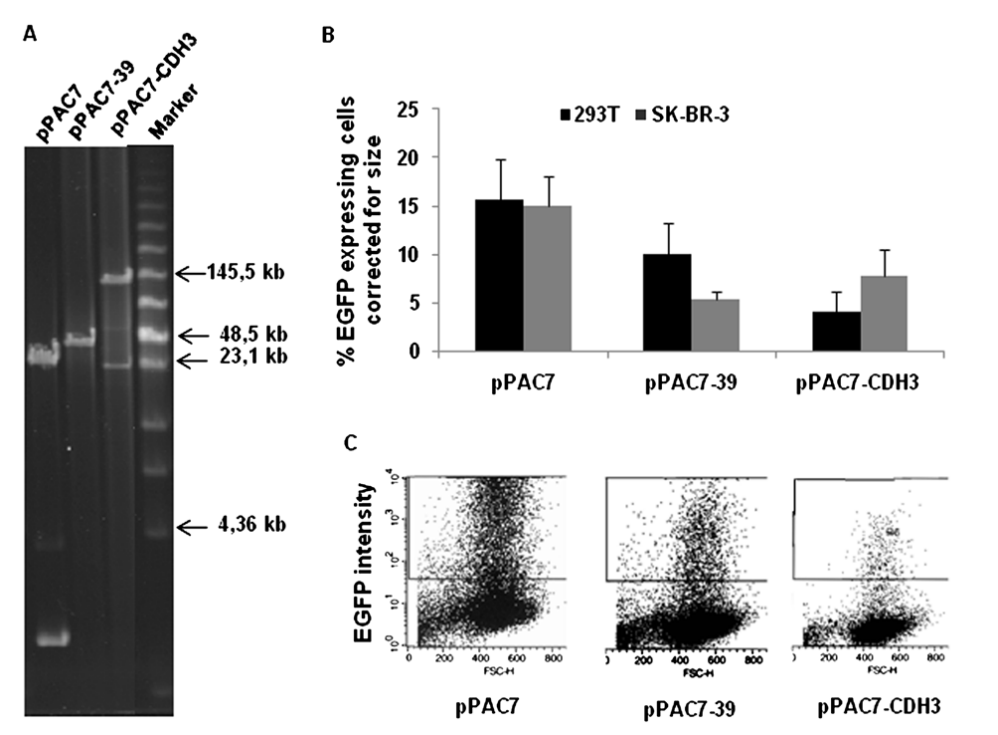
**Transfection efficiencies measured in 293T and SK-BR-3 cells**. A) DNA from each of the constructs pPAC7, pPAC7-39 and pPAC7-CDH3 was digested with *Not*I and separated on a BioRad CHEF-mapper. During construction of pPAC7-39 one of the *Not*I restriction sites got deleted and thus giving a linearised fragment when digested with *Not*I. Selected fragments in the Low Range PFG Marker are indicated. B) Diagram showing the fraction of viable, EGFP-expressing 293T and SK-BR-3 cells measured by flow cytometry the day after the transfection with the following constructs: pPAC7, pPAC7-39 and pPAC7-CDH3. One μg of DNA from each construct was used for transfection, and the figure shows the transfection frequencies normalized with respect to the molar concentrations of plasmid DNA used in each transfection. For the 293T cell line, each bar represents three independent experiments all performed in triplicate. For the SK-BR-3 cell line each bar represents two separate experiments, both performed in triplicates. Standard deviations are indicated. C) Representative flow diagrams showing a 10-fold decrease in EGFP intensities in the 293T cell line with increasing plasmid size. Constructs used are: pPAC7, pPAC7-39 and pPAC7-CDH3.

There is conflicting evidence on whether construct size directly affects the efficiency of transfection into mammalian cells [32-35]. Using pPAC7, pPAC7-39 and pPAC7-CDH3 provided us with plasmids in a size range from 23,4 kb to 155 kb (Figure 4A), all expressing the EGFP reporter gene under the control of the same promoter. These constructs were transfected into 293T and SK-BR-3 cells, and the transfection efficiencies were directly measured by flow cytometry.

A decreasing fraction of EGFP positive cells were detected with increasing construct size (results not shown). However, since the DNA mass is a critical parameter for transfections, a fixed mass of DNA from each construct was used in these experiments. When the transfection efficiencies are normalized with respect to the molar concentrations of plasmid DNA (Figure 4B), a decrease in transfection efficiency with increasing plasmid size was observed in 293T. However, this tendency was not distinct in SK-BR-3. The decreased transfection efficiency with increasing plasmid size detected in 293T might be due to the smaller number of plasmid molecules delivered to the cells when transfecting large plasmids compared to smaller ones. This is in agreement with McLenachan and co-workers [35] who concluded that cellular uptake is largely independent of DNA size.

In the present work the transfected cells showed decreased fluorescence intensities with increasing plasmid size (Figure 4C). This is in agreement with previous observations that cells successfully transfected by larger plasmids show lower level of marker gene expression than those transfected with smaller plasmids [33,34]. It has been suggested that large plasmids are more susceptible to degradation within the cell because the mobility through the cytoplasm decreases with increasing plasmid size [35]. Thus decreased marker expression with increasing plasmid size could be a result of lower copy number in the nucleus of cells transfected with the large constructs. This effect may also explain the decrease in luciferase levels in the cell populations previously reported by Kreiss and co-workers [32]. In conclusion, our results support the hypothesis that cellular uptake of large DNA constructs in human cell lines is not only dependent on DNA size.

## Conclusion

We present an EGFP reporter PAC shuttle vector, pPAC7, which can serve as an important tool in functional studies, one application being validation of candidate disease genes. After introduction of a 132 kb fragment containing the complete *CDH3 *gene in its native genome context, we show regulated expression of the gene carried on the construct. We also performed transfection experiments with a series of vectors in the size range 23,4 kb to 155 kb, and detected no apparent correlation between construct size and transfection efficiency.

## Methods

### Vector construction

This report describes a bacterial artificial chromosome vector, pPAC7 [GenBank: FJ710109], which was constructed from two precursor vectors, pE106 and pPAC3.2, used in the construction of pPAC4 [28]. The plasmid pE107 was made by replacing the hCMV-cassette from pE106 with an adapter (ctagtgtcgactttacgcgtg), introducing unique *Sal*I and *Mlu*I sites between the *Spe*I and *Bst*EII sites in pE106. The *EBNA-1 *gene was obtained as a *Spe*I fragment from the vector pEBAC160^2-3 ^(kindly provided by Dr. Panos Ioannou, The Murdoch Children's Research Institute) and cloned into pE107, resulting in the vector pE108. pE108, containing *EBNA-1*, the *Bsr *Cassette, and the lambda cos site, was digested with *Nhe*I and *Bst*EII and ligated to *Nhe*I/*Bst*EII digested pPAC3.2. The clone containing the correct sequence elements was named pPAC5.

The *EGFP *gene was first transferred from the pEGFP-C1 vector (Clontech Laboratories, Palo Alto, CA, USA) into pE103 which already contained the *ori*P fragment, *att*Tn7, *loxP*^511 ^and the PI-SceI site [28]. Then a *Bss*HII-fragment from pE103-EGFP containing the *EGFP *and *ori*P genes was ligated to a pPAC5 *Asc*I fragment. A clone containing the correct sequence elements was named pPAC7. All cloning intermediates were analyzed by restriction digests and sequenced using the appropriate primers [see Additional file 2].

### Cloning the complete *CDH3 *gene into pPAC7

The PAC clone RPCI6-77A18 has previously been shown to contain the complete *CDH3 *gene [36]. The 132 kb insert in this clone was transferred to the vector pPAC7 as a *Not*I-fragment. Colonies obtained in this cloning step were screened by PCR [see Additional file 1]. Positive clones were analyzed by PFGE and sequencing over the cloning junctions [see Additional file 1]. The construct containing the complete 132 kb insert was named pPAC7-CDH3 (see Figure 2). A smaller PAC-construct was also made in this cloning process by transferring a 18,5 kb *Not*I fragment into pPAC7. The size of this construct, named pPAC7-39, was verified by PFGE.

### Growth and transfection of mammalian cells

The 293T cell line (ATTC CRL 11268), which is an adenovirus transformed primary human embryonic kidney cell line expressing the SV40 large T antigen, and the human mammary adenocarcinoma cell line, SK-BR-3 (ATTC HTB-30) and its derivatives were grown in RPMI1640 with phenol red (Bio Whittaker, MD, USA) supplemented with 2 mM L-glutamine and 10% FBS (Gibco BRL^®^, CA, USA). For experiments related to the functional studies of CDH3, the human mammary adenocarcinoma cell lines MCF-7, SK-BR-3 and its derivatives, the human mammary ductal carcinoma cell line ZR-75-1, 293T and 293E (293T stably expressing the Epstein-Barr virus nuclear antigen), were grown in RPMI1640 without phenol red (Gibco BRL^®^, CA, USA) supplemented with 25 mM Hepes (Gibco BRL^®^, CA, USA), 10% charcoal-stripped FBS (Innovative Research, MI, USA) and 2 mM L-Glutamine (Gibco BRL^®^, CA, USA).

Transfections were performed using a ratio of 10:4 of the Lipofectamine 2000 (Invitrogen, CA, USA) reagent (μl) to DNA (μg). Cells were plated in 6 well plates at a density of 6 × 10^5 ^cells per well, 24 hours prior to transfection. All experiments were performed in triplicate. In experiments where selection was applied, medium containing 2, 5 and 10 μg/ml blasticidin (InvivoGen CA, USA) was added 2 days after transfection of the SK-BR-3 cell line.

Pictures of transfected cells were taken using a Zeiss Axiovert 200 M microscope, and the data processed using the Axio Vision 3.1 program. Black and white pictures showing the cell population and pictures showing the fluorescent cells were compared.

### Flow cytometry

Flow cytometry was performed using a Becton Dickinson dual-laser FACScalibur analysis instrument (BD Biosciences, CA, USA) and the Cell Quest software. Cells were harvested 24 hours after transfection by adding trypsin-EDTA. The cell suspension (500 μl) was mixed with a pipette and filtered through a filter cloth (50 μm, Eiolf Thon) into Falcon sample tubes. 1 μl propidium iodide (1 mg/ml) was added to the cell suspensions before the samples were analyzed.

Cell sorting was performed using a Becton Dickinson triple-focus Diva. The samples were prepared as described for flow cytometry. The sorted cells were plated in a 12 well plate.

### Relative quantification of CDH3 expression

Total RNA from cells harvested in 1× Nucleic Acid Purification Lysis Solution (Applied Biosystems, Foster City, CA, USA) was extracted on the ABI PRISM^® ^6100 Nucleic Acid PrepStation (Applied Biosystems, Foster City, CA, USA) using the Cultured Cells kit (Applied Biosystems, Foster City, CA, USA). The RNA was reverse transcribed using the High Capacity cDNA Archive Kit (Applied Biosystems, Foster City, CA, USA), and cDNA synthesis was performed on a Gene Amp^® ^PCR system 9700 (Applied Biosystems, Foster City, CA, USA), programmed with an initial step at 25°C for 10 minutes, followed by 37°C for 2 hours and 85°C for 5 seconds.

For real-time quantification of relative gene expression, Applied Biosystems' ABI PRISM 7900 HT Sequence Detection System was used at the following conditions: 50°C for 2 minutes, 95°C for 10 minutes followed by 40 cycles with 95°C for 15 seconds and 60°C for 1 minute. All reagents were purchased from Applied Biosystems; TaqMan Universal PCR Master Mix and the TaqMan Gene Expression Assays: CDH3 (Assay ID: Hs_00999916_m1), and PMM1 (Assay ID: Hs_00963625_m1). The quantification of cDNA was performed using the comparative CT method (Livak and Schmittgen, 2001), where the amplification of the target (CDH3) and endogenous control (PMM1) was run in separate tubes. The amount of target was calculated by normalization to an endogenous control, relative to a calibrator sample (Stratagene^® ^QPCR Human Reference Total RNA, Cat. No. 750500, Stratagene, La Jolla, CA, USA).

## Authors' contributions

HAA performed the majority of the experiments and drafted the manuscript. EG contributed in flow cytometry and transfection experiments. GLS constructed some vector intermediates and contributed in the transfection experiments. MML and JT contributed in real time experiments and large insert cloning, respectively. HP and EF provided supervision. EF coordinated the project and contributed in the manuscript preparation. All authors read and approved the final manuscript.

## Supplementary Material

Additional file 1 **Supplemental Table 1**. Genomic PCR primers used to verify the deletion covering the entire CDH3 gene in SK-BR-3. Click here for file

Additional file 2 **Supplemental Table 2**. Sequencing primers used to verify cloning steps in the construction of pPAC7. Click here for file
